# Individual-based approach to epidemic processes on arbitrary dynamic contact networks

**DOI:** 10.1038/srep31456

**Published:** 2016-08-26

**Authors:** Luis E. C. Rocha, Naoki Masuda

**Affiliations:** 1 Department of Mathematics and naXys, Université de Namur, 8 Rempart de la Vierge, B-5000 Namur, Belgium; 2 Department of Public Health Sciences, Karolinska Institutet, 18A Tomtebodavägen, S-17177 Stockholm, Sweden; 3 Department of Engineering Mathematics, University of Bristol, Woodland Road, Bristol BS8 1UB, United Kingdom

## Abstract

The dynamics of contact networks and epidemics of infectious diseases often occur on comparable time scales. Ignoring one of these time scales may provide an incomplete understanding of the population dynamics of the infection process. We develop an individual-based approximation for the susceptible-infected-recovered epidemic model applicable to arbitrary dynamic networks. Our framework provides, at the individual-level, the probability flow over time associated with the infection dynamics. This computationally efficient framework discards the correlation between the states of different nodes, yet provides accurate results in approximating direct numerical simulations. It naturally captures the temporal heterogeneities and correlations of contact sequences, fundamental ingredients regulating the timing and size of an epidemic outbreak, and the number of secondary infections. The high accuracy of our approximation further allows us to detect the index individual of an epidemic outbreak in real-life network data.

Infectious diseases are a major concern of public health because of the potentially high mortality and financial costs to health systems^1^. To avoid or reduce the impact of an epidemic outbreak, it is necessary to understand the mechanisms driving the spreading dynamics. The population dynamics of infectious diseases depends on the particular pathogen and on the transmission routes between individuals. Airborne infections, including influenza and tuberculosis, may spread through close contacts between a host and a susceptible individual. Sexual contacts on the other hand create the main route for the spread of infections such as HIV and chlamydia^2^. Various forms of daily interactions among people thus form complex contact networks that define the potential infection routes^3^,^4^. These contact networks are characterised by different patterns of connectivity between the individuals and by the timings of the contact events^3^,^5^. Previous research has provided substantial understanding of the importance of the structure of contacts (e.g., clustering, number of contacts^6^,^7^,^8^) and of temporal correlations (e.g., contact times, concurrency^9^,^10^) to regulate the spread of infectious diseases. However, how structural and temporal properties compete and synergistically change spreading dynamics remains not sufficiently understood^11^,^12^,^13^.

Given the complexity and heterogeneity of contact networks in general, a dominant approach to study epidemics on dynamic networks is to numerically simulate a stochastic epidemic process. Theoretical approaches however may be useful for further mechanistic understanding of epidemic dynamics and for developing efficient intervention protocols in principled ways. There are several lines of such theoretical approaches^8^. Among them is the individual-based approximation (IBA), also termed discrete-time Markov chains, which is applicable to arbitrary contact networks. The key idea of the IBA is tracking the dynamics of the probability that an individual is in a certain state (e.g., infected state). The IBA has been applied on static networks to study susceptible-infected-susceptible (SIS)^8^,^14^,^15^,^16^,^17^,^18^ and susceptible-infected-recovered (SIR)^8^,^19^,^20^,^21^,^22^ epidemic models. Although the applicability of the IBA is hampered by correlation between the states of different nodes, in particular between adjacent nodes^8^,^20^,^21^, the same technique applied to dynamic contact networks may be rather accurate because the dynamics of contact networks may decorrelate the states of different nodes. Individual-based methods have been applied for understanding the physics of the SIS epidemics on dynamic networks, in combination with synthetic adaptive networks^23^ and network data extended with the temporal periodic boundary condition^24^.

In the present study, we develop the IBA for the susceptible-infected-recovered model on dynamic contact networks as observed on real settings. Our model describes a broad class of infectious diseases, such as measles, chickenpox, and Ebola, where hosts develop immunity or die after a given infectious period^2^. We use our framework to estimate the dynamics of macroscopic epidemiological variables such as the time-dependent prevalence of infections, formulate the effective reproduction number (i.e., the number of secondary infections produced by a single infected individual in a finite population^25^), quantify super-spreaders^26^, and detect the source of infections if past contacts and the epidemiological state of the population are known at a given time^27^.

## Results

### Dynamic Contact Networks

A dynamic contact network is defined as a sequence of snapshots. A snapshot is a contact network of *N* nodes (i.e., individuals) represented by a contact matrix ***A***(*t*) = (*a*_*ij*_(*t*)) (1 ≤ *i*, *j* ≤ *N*, 1 ≤ *t* ≤ *t*_max_). We set *a*_*ij*_(*t*) = 1 if a link (i.e., a contact) (*i*, *j*) exists at time *t* and *a*_*ij*_(*t*) = 0 otherwise. We assume that each snapshot is an undirected network such that ***A***(*t*) is a symmetric matrix. Each snapshot corresponds to time *T*_w_, representing the temporal resolution of observation of contact networks or the level of coarse graining of contact networks in terms of time. If the total observation time of a contact network is *T*, the number of snapshots is equal to *t*_max_ ≡ *T*/*T*_w_. The framework of dynamic networks is relevant if *T*_w_ is smaller than or comparable with the time scale of the epidemics process. Otherwise, the network changes more slowly than the epidemic states, and the static network approximation is sufficient. In the present paper, we study high-resolution contact network data with *T*_w_ = 20 sec, which is much smaller than the time scale of real epidemic processes, in which the infectious period typically lasts a few days or more^28^.

### SIR Model on Dynamic Networks

We consider the discrete-time SIR model in which a snapshot network corresponds to a single time step. At each time, an individual is either in the susceptible (S), infected (I), or recovered (R) state. Each individual experiences at most one state-transition event within a snapshot. This assumption is valid when *T*_w_ is sufficiently small relative to the time scale of the SIR dynamics. Upon contact, an infected individual infects a susceptible with (per-contact) probability *β*. An infected individual recovers with probability *μ* in each snapshot. We assume that the recovery events occur prior to the infection events in each snapshot. This assumption is reasonable if *T*_w_ is sufficiently small.

### Individual-based Approximation

We denote by *S*_*i*_(*t*), *I*_*i*_(*t*), and *R*_*i*_(*t*) the probability that individual *i* is in the state S, I, and R at time *t*, respectively; therefore, *S*_*i*_(*t*) + *I*_*i*_(*t*) + *R*_*i*_(*t*) = 1. The probability *p*_*ij*_(*t*) that individual *i* is not infected by individual *j* at time *t*, under the condition that *i* is in state S at time *t* − 1, is given by


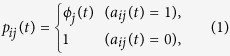


for *t* ≥ 1, where


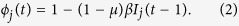


If there is no contact between *i* and *j* at time *t*, *i* is not infected by *j* at this time *t* such that *p*_*ij*_(*t*) = 1. Otherwise, *j* infects *i* if and only if *j* is infected (with probability *I*_*j*_(*t* − 1)), *j* does not recover at time *t* (with probability 1 − *μ*), and the infection occurs with probability *β* (equation (2)). Note that *p*_*ij*_(*t*) is independent of *i*.

Equation (1) is supplied with


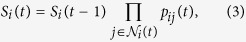



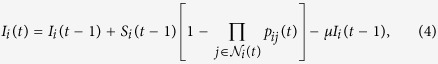



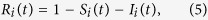


where the set of neighbours of the individual *i* at time *t* is denoted by 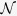
_*i*_(*t*) ≡ { *j*; *a*_*ij*_(*t*) = 1}. Equation (3) and equation (4) are only approximate because the expression 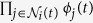 assumes that *I*_*j*_(*t*) for different *j* values represents independent events. In fact, the states of different individuals are generally correlated. For example, the true probability that two individuals *i* and *j* are simultaneously infected at time *t* may be larger or smaller than *I*_*i*_(*t*) *I*_*j*_(*t*). It is straightforward to extend the IBA to the case of weighted networks. The IBA is known for the continuous-time SIR model on static networks^19^,^20^,^21^,^22^. Adapting it to the case of dynamic networks and discretising the time yield a set of equations similar to equations (1, 2, 3, 4, 5) (see Supplementary Information).

To calculate the IBA in each time step, we start by calculating *ϕ*_*j*_(*t*) (1 ≤ *j* ≤ *N*), which requires *O*(*N*) time. Then, we scan the list of contacts at time *t*, by which we can calculate the most time-consuming part, i.e., 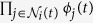. This operation requires *O*(*N* 〈*k*〉_snap_) time, where 〈*k*〉_snap_ is the mean number of contacts per individual in a snapshot. Therefore, running the IBA for the entire dynamic network data requires *O*(*N* 〈*k*〉_snap_ *t*_max_) time. Running a direct numerical simulation of the SIR dynamics consumes *O*(*N*) time for possible recovery events and *O*(*N* 〈*k*〉_snap_) time for possible infection events for each time step. Therefore, the total time for a single realisation is of the same order as that for the IBA. The merit of the IBA is thus that it tracks the evolution of the probability, corresponding to infinitely many realisations of direct numerical simulations, with the same order of the computation time.

### Accuracy of the Individual-based Approximation

We calculate *S*_*i*_(*t*), *I*_*i*_(*t*), and *R*_*i*_(*t*) (1 ≤ *i* ≤ *N*) in increasing order of time *t* from *t*_1_ = 0 to *t*_2_ = *t*_max_. We use each *i* (1 ≤ *i* ≤ *N*) as the index individual such that the initial condition of the IBA is given by *S*_*j*_(0) = 1 − *δ*_*ij*_, *I*_*j*_(0) = *δ*_*ij*_, and *R*_*j*_(0) = 0, 1 ≤ *j* ≤ *N*, where *δ* is the Kronecker delta. The expected fraction of infected individuals (i.e. prevalence) at time *t* is given by 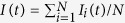. Similarly, the expected fractions of susceptible and recovered individuals at time *t* are given respectively by 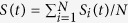 and 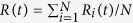.

**Figure 1:**
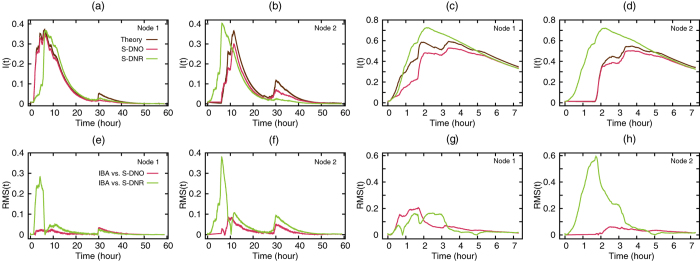
Accuracy of different approximation methods. (**a–d**) The fraction of infected individuals as a function of time (i.e. the prevalence *I*(*t*)) for *β* = 0.1 and *μ* = 0.001 using the (**a,b**) conference data set, and (**c,d**) museum data set for a given seed individual. Root-mean-square deviation between (**e,f**) IBA and S-DNO, and (**g,h**) IBA and S-DNR.

In the following, we describe the results for the conference and museum face-to-face network data sets^29^ (see Materials and Methods). The results for a third data set (in a hospital context) are qualitatively the same (see Supplementary Information). Figure 1(a–d) show the evolution of *I*(*t*) for two arbitrarily selected index individuals for each network, node 1 and node 2. In the figure, the estimate by the IBA is compared with that obtained from two other approaches: (i) direct numerical simulations on the original dynamic network, abbreviated as S-DNO, and (ii) direct numerical simulations on the random network in which links within a day are randomised but the daily cycles and network structure are conserved, abbreviated as S-DNR (see Materials and Methods for the precise definition). We compare the IBA with S-DNO to estimate the performace of our theoretical approximation against direct simulation on the empirical network data. We also compare the IBA with S-DNR to show that the IBA captures non-random temporal structures in the contact patterns. To calculate *I*(*t*) for S-DNO and S-DNR, we average over 200 realisations of the simulation. However, we exclude the outbreaks in which less than 0.1*N* nodes have finally experienced the infected state, i.e., minor outbreaks in which the index individual has infected no other, or a small fraction, of individuals in the entire observation interval. We exclude these near-null outbreaks because approximations using probabilistic flows, including the IBA, are generally accurate under the condition that minor outbreaks are eliminated^20^.

The figure indicates a generally good agreement between the IBA and direct simulations on the dynamic network (S-DNO). The IBA captures the effect of variations in the contact patterns within and between multiple days. For example, we observe a second wave of infections after the first wave has decayed to low levels in the conference data (Fig. 1(a,b)). The IBA also captures the absence of epidemics in the first two hours on the museum network when node 2 is initially infected (Fig. 1(d)). The fact that multiple waves and sudden changes in *I*(*t*) are observed for simulations averaged over 200 realisations indicates that they are phenomena shared by a majority of realisations yielding non-null outbreaks. The waves result from temporal patterns of the networks, i.e., concentration of contacts around certain times of the day and the absence of contacts during night.

The approximate dynamics obtained from the S-DNR is also presented in Fig. 1(a–d). We observe that the prevalence is either under- or overestimated depending on the node and data set. This result reflects the fact that contacts are now uniformly distributed during the days in contrast to the real patterns that contain, for example, temporally clustered activitiy. For the same simulations, we measure the root-mean-square deviation in time RMS(t) between the IBA and S-DNO and between the IBA and S-DNR in Fig. 1(e–h). The IBA more accurately reproduces the spread on the original network data (S-DNO) than on the randomised networks (S-DNR).

**Figure 2:**
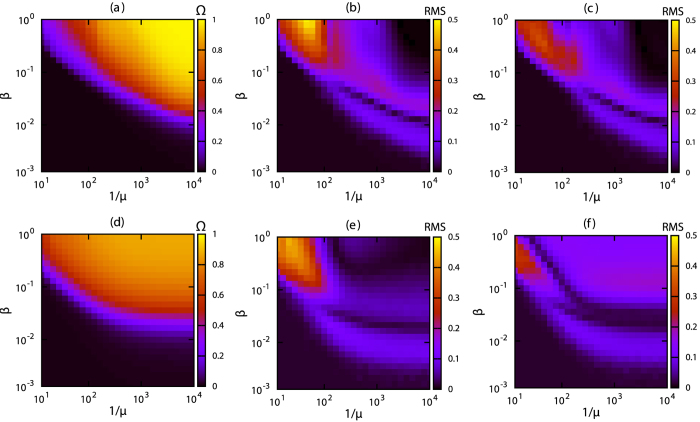
Final outbreak size. (**a,d**) Final outbreak size Ω ≡ *R*(*t*_max_) + *I*(*t*_max_) for the IBA. We take averages over all individuals as seeds. Root-mean-square deviation (RMS) between (**b,e**) the IBA and S-DNO and (**c,f**) the IBA and S-DNR. (**a–c**) correspond to the conference data set and (**d–f**) to the museum data set. For the S-DNO and S-DNR cases, we remove null and small (<0.1*N*) outbreaks to calculate Ω. The colours represent (**a,d**) the values of Ω and (**b**,**c**,**e**,**f**) the root-mean-square difference between the two approaches.

The IBA yields an increase in the final outbreak size Ω ≡ *R*(*t*_max_) + *I*(*t*_max_) for increasing values of *β* and decreasing values of *μ* for both data sets (Fig. 2(a,d)). In a wide region of the *β*-*μ* parameter space, the final outbreak size for the S-DNO is accurately estimated by the IBA for both network data sets, particularly for small *μ* (Fig. 2(b,e)). The accuracy of the IBA degrades for large *μ* because the chance of generating no secondary infections in the simulations increases. Results for the S-DNR model (Fig. 2(c,f)) are similar to those for the S-DNO (Fig. 2(b,e)) although the IBA overestimates Ω for the S-DNR for the museum data set when *β* is large and *μ* is small (Fig. 2(f)). These results are consistent with those shown in Fig. 1(a–d) and reinforce the fact that the IBA better reproduces S-DNO.

### Super-spreading

Hosts that infect disproportionally more secondary contacts than the average are known as super-spreaders. Super-spreading is observed in a range of infectious diseases such as sexually transmitted infections, SARS, and smallpox^26^,^30^ and is not simply determined by the number of contacts that an individual owns but significantly by its position in the contact networks^31^. Identifying super-spreaders is a fundamental step towards efficient infection interventions because targeting super-spreaders potentially saves resources^26^. We therefore define the individual effective reproduction number^30^,^32^ *R*_eff_(*i*, *t*_1_, *t*_2_) for dynamic contact networks as


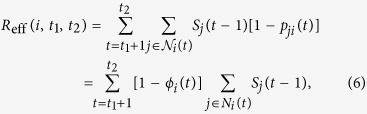


where the epidemic process starts at time *t* = *t*_1_ with the sole infected individual *i*, and *t*_2_ is the ending time of the observation. The individual effective reproduction number takes into account the fact that in finite populations, which is the focus of the present study, some individuals may be infected by others before having a chance to be infected by the index individual. We calculate the number of secondary infections caused by *i* between times *t*_1_ + 1 = 1 and *t*_2_ = *t*, and thus abbreviate *R*_eff_(*i*, *t*_1_, *t*_2_) as *R*_eff_(*i*, *t*).

**Figure 3:**
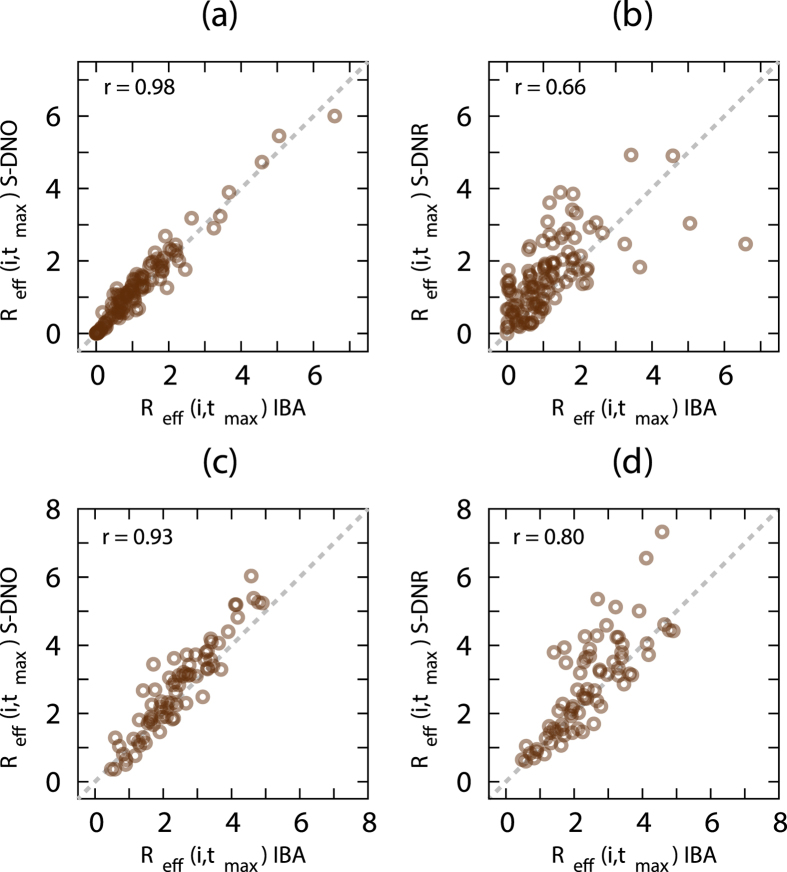
Individual effective reproduction number. Comparison between the IBA and S-DNO for the (**a**) conference and (**c**) museum data sets. Comparison between the IBA and S-DNR for the (**b**) conference and (**d**) museum data sets. Each of the *N* = 113 circles (conference) and *N* = 72 (museum) the N circles (N = 113 and N = 72 for the conference and museum data, respectively) represents an index individual *i*. Values are estimated at time *t*_max_. We set *β* = 0.1 and *μ* = 0.001. The Pearson correlation coefficient is denoted by *r*.

For one pair of *β* and *μ* values, *R*_eff_(*i*, *t*_max_) estimated by the IBA is plotted against that calculated by the S-DNO for the conference (Fig. 3(a)) and museum (Fig. 3(c)) data sets. Each circle in the figure represents an index individual *i*. The values for the S-DNO represent the number of individuals that *i* has actually infected, averaged over 200 realisations of the simulation. We first note that the IBA estimates *R*_eff_(*i*, *t*_max_) obtained from the S-DNO reasonably well, with a large value of the Pearson correlation coefficient *r*. The IBA is less accurate at approximating *R*_eff_(*i*, *t*_max_) obtained from S-DNR (Fig. 3(b,d)). The infection potential is also highly heterogeneous across individuals. In both IBA and S-DNO, a few individuals cause much more secondary infections (that is, the super-spreaders) than the majority of the individuals that typically infects only a few other individuals.

### Effective Reproduction Number

**Figure 4:**
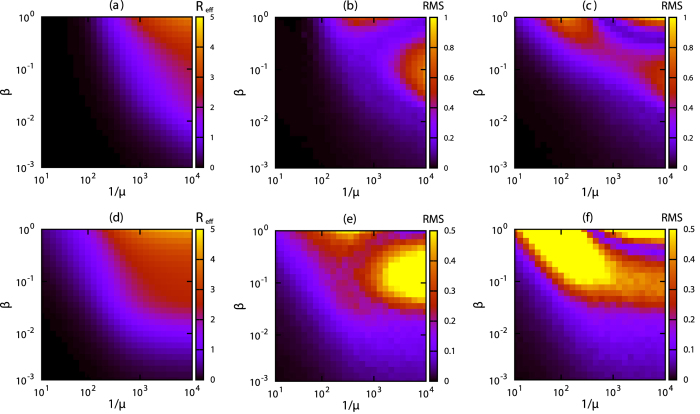
Effective reproduction number. Estimation of the effective reproduction number *R*_eff_(*t*_max_) using the IBA for (**a**) the conference and (**d**) museum data sets. Root-mean-square (RMS) deviation between the IBA and S-DNO for (**b**) the conference and (**e**) museum data sets, and between the IBA and S-DNR for (**c**) the conference and (**f**) museum data sets for various values of the epidemiological parameters *β* and *μ*. The colours in (**a,d**) represent the value of *R*_eff_ (*t*_max_) and in (**b,c,e,f**) the root-mean-square deviation. See Materials and Methods for details on the numerical simulations.

The basic reproduction number *R*_0_, defined as the expected number of secondary infections caused by an index infected individual in a fully susceptible population, is typically used as a threshold to characterise the potential of an epidemic outbreak in the population. The epidemics is likely to occur if and only if *R*_0_ > 1 2,33. The reproductive number is a key quantity connected to, for example, the outbreak size and herd-immunity^34^. An accurate estimate of *R*_eff_ is thus necessary to properly assess the effectiveness of public health interventions. We define the effective reproduction number, *R*_eff_(*t*), which generalises *R*_0_ to be time-dependent and takes into account that the population is not fully susceptible as the time progresses. Using equation (6), we define *R*_eff_(*t*) as the average number of secondary infections caused by a single index individual, i.e., 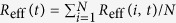. For a range of parameters *β* and *μ*, *R*_eff_(*t*_max_) estimated using the IBA gives a relatively low average number of secondary infections (Fig. 4(a,d)). The IBA estimation agrees (i.e., small root-mean-square deviation) with that obtained from direct numerical simulations, i.e., S-DNO (Fig. 4(b,e)), particularly for the conference data set. For the museum data set, the error is relatively large for some combinations of parameters. The S-DNR agrees relatively well with the IBA for the conference data set (Fig. 4(c)) but not for the museum data set (Fig. 4(f)).

### Importance of Early Times

Human interaction typically generates strong heterogeneity in temporal contact patterns. The order of the contacts generally regulates the speed and size of an epidemic outbreak since very active individuals may spread the infections quicker than others^35^,^36^.

**Figure 5:**
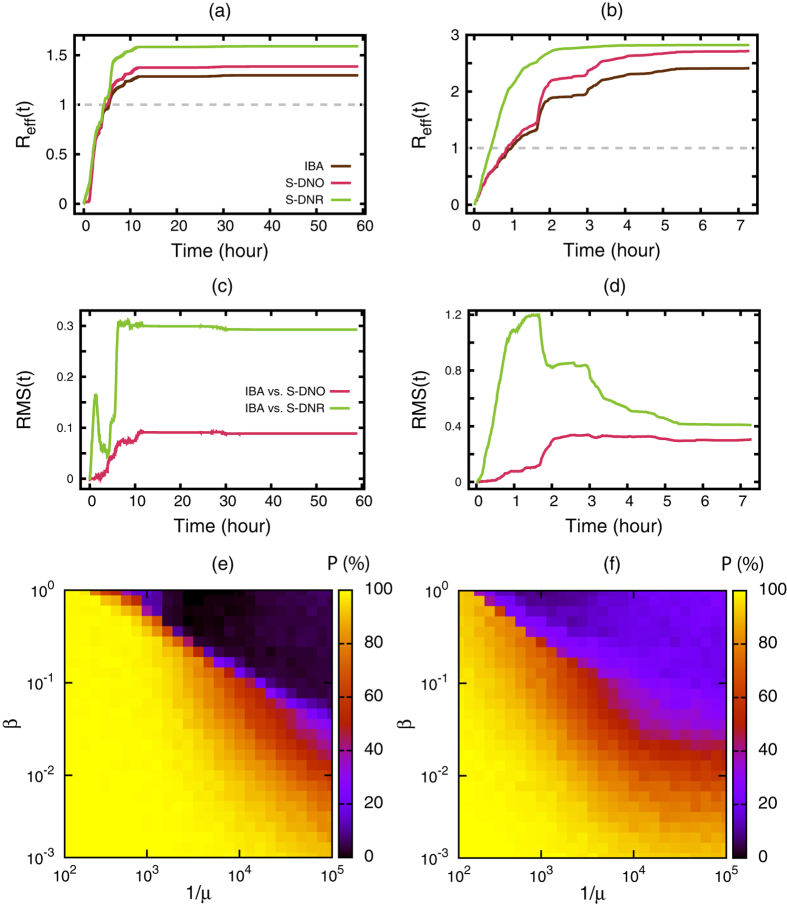
Time dependence of the effective reproduction number and source detection. Time dependence of the effective reproduction number *R*_eff_(*t*) calculated using different models with *β* = 0.1 and *μ* = 0.001 for the (**a**) conference and (**b**) museum data sets. Root-mean-square difference between the IBA and S-DNO and IBA and S-DNR for the (**c**) conference and (**d**) museum data sets. See Materials and Methods for details on the calculations. Percentage (P) of successfully detected infection sources (estimated on the basis of 1000 randomly located sources) for various *β* and *μ* values for the (**e**) conference and (**f**) museum data sets.

Time courses of the effective reproduction number, *R*_eff_(*t*), for the two data sets are shown in Fig. 5(a,b). Figure 5(b) shows that *R*_eff_(*t*) irregularly increases in time if the original dynamic contact network is taken into account (i.e., IBA and S-DNO). The sudden jumps correspond to the periods in which contacts are dense, a behavior lost in the randomised version of the network. We see that *R*_eff_(*t*) converges within a day, indicating that only early contacts influence the final effective reproduction number, *R*_eff_(*t*_max_). The IBA (brown lines in Fig. 5(a,b)) reproduces the time course of *R*_eff_(*t*) obtained from the S-DNO (red lines) with a reasonable accuracy. Estimation of *R*_eff_(*t*) on the basis of S-DNR (green line in Fig. 5(a,b)) also shows saturating behaviour but overestimates *R*_eff_(*t*) obtained from the S-DNO for both data sets. Quantitatively, we observe a small deviation favoring the S-DNO framework (Fig. 5(c,d)). Our results suggest that as far as *R*_eff_(*t*_max_) is concerned, it does not help to sample contact patterns for long times to improve estimation. This reasoning depends on the values of *β* and *μ*; if *β* or *μ* is very small, it takes long time for the epidemics to take off or to get extinguished. Under such conditions, relatively late snapshots may influence *R*_eff_(*t*_max_).

### Estimation of Source of Infection

Finding the index case (or patient zero) helps to understand how an infection has been introduced in the population and to trace transmission trees^37^. The increasing availability of network data has motivated the development of algorithms to detect the source of epidemic spreading on contact networks^38^,^39^,^40^. Here we consider the problem of inferring the source of an epidemics when only the information about the current state of each individual and past contact patterns is available^27^. Our approach is realistic in the context of schools or hospitals for example, where contact patterns may be monitored^41^,^42^. We first assume that we can observe the state of all individuals at a given time *t*. We then set a Boolean variable (i.e. 1 if a given state is observed and 0 otherwise) to describe the state of each individual *i* as susceptible, *N*(*S*,*i*), infected, *N*(*I*, *i*), or recovered, *N*(*R*, *i*) (1 ≤ *i* ≤ *N*), such that *N*(*S*, *i*) + *N*(*I*, *i*) + *N*(*R*, *i*) = 1 at a given time (the time variable is suppressed). The aim is to infer the most likely source of infection 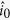 given a configuration of *N*(*S*, *i*), *N*(*I*, *i*), *N*(*R*, *i*) (1 ≤ *i* ≤ *N*) at time *t*, and the contact sequence ***A***(*t*^′^) (*t*^′^ = 1, …, *t*).

Using the IBA, we then calculate *S*_*i*_(*t*), *I*_*i*_(*t*), and *R*_*i*_(*t*) (1 ≤ *i* ≤ *N*) for each source individual *i*_0_ (1 ≤ *i*_0_ ≤ *N*). For example, *S*_*i*_(*t*) is interpreted as the probability that a single realisation yields a configuration at time *t* such that node *i* is susceptible (remember that *S*_*i*_(*t*) + *I*_*i*_(*t*) + *R*_*i*_(*t*) = 1). The IBA assumes that the states of different individuals are independent of each other. Therefore, the probability that *N*(*S*,*i*), *N*(*I*,*i*), and *N*(*R*,*i*) (1 ≤ *i* ≤ *N*) are attained is given by


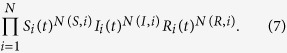


The most likely source of infection 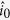 is the *i*_0_ value that maximises equation (7), i.e.


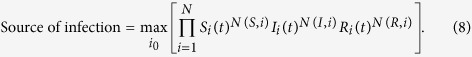


To test our algorithm, we set *t* = *t*_max_ and create an epidemic scenario by numerically simulateing a single stochastic epidemic outbreak for a pair *β* and *μ* on the contact sequence, starting from an infected individual *i*_source_ at time *t* = 0. Figure 5(e,f) shows that the performance of this algorithm is relatively good for both data sets. The fraction of the correct estimation (i.e., 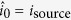) is above 20% for most combinations of *β* and *μ*. As a comparison, random success would be 1/113~0.8% and 1/172~1.4% for the conference and museum data sets, respectively. Our results are as good as the results obtained by methods using statistical inference^27^. In the case of the conference data set, the performance of the successful detection degrades for large infection probabilities (e.g., *β* > 0.1) and long infection periods (e.g., 1/*μ* > 1000~5.55 hours), a regime in which almost the entire population gets infected (Fig. 5(e)). This decrease in performance is not observed for the museum data set (Fig. 5(f)). In contrast, the source detection is successful in more than 80% of the cases (Fig. 5(e,f)) if the size of the epidemics is not too large (Fig. 2).

## Discussion

The occurrence of epidemic outbreaks typically depends on the pathogen and contact patterns between hosts and susceptible individuals. Previous studies emphasised the importance of the structure of the contact networks on the infection dynamics. The increasing availability of high-resolution longitudinal data has shown, however, that contact patterns are also dynamic across various time scales. A single framework, able to capture all these structural and temporal patterns, is thus necessary to fully understand the population dynamics of infectious diseases.

In this paper, we presented the individual-based approximation (IBA) to model SIR epidemics on arbitrary dynamic contact networks. The IBA neglects the correlations between the states of different individuals, most significantly between neighbouring network nodes. For example, it misses the fact that an individual is more likely to be infected if its neighbour is infected and vice versa. The correlation would develop for networks with a small mean number of contacts and high clustering (i.e., three nodes forming a connected triangle). In such a case, the pair approximation, which explicitly tracks the evolution of the probabilities of pairwise states (the states of adjacent pairs of individuals) at a mean field level^6^,^8^, or an extension of the IBA to account for pairwise correlation^20^,^21^ is more accurate. The concept of pairwise correlation is less straightforward if nodes and links appear and disappear in time. The temporality of the network may effectively decorrelate the state of the individuals, which may be why the IBA is relatively accurate at approximating the results obtained from direct numerical simulations.

The IBA showed good performance to estimate the final outbreak size and the reproduction number on real-life dynamic contact networks. These results indicate that the timings of contacts cannot be discarded if one wants to estimate epidemic outbreaks. Our results further suggest that only relatively short intervals of contact network data are necessary to estimate the reproduction number. More importantly, we showed that if longitudinal network data were available, the source of an epidemic could be efficiently detected, assuming an SIR dynamics, even if only the current state of the epidemics was known. These illustrative applications of the IBA may be used for surveillance in closed environments, such as schools^41^ and hospitals^42^, where protocols to collect human interaction data are already available and outbreaks of infectious diseases may have major consequences. Furthermore, since the IBA framework provides a principled way to calculate probability flows with a single sweep of a given contact network, it allows a computationally efficient estimation of the most-likely transmission trees on large dynamic networks. This information can be exploited to design efficient strategies for infection control such as immunisation^43^ and travel restrictions^44^,^45^, or to identify sentinels for early detection of epidemics^46^,^47^.

To calculate the prevalence for the numerical simulations on dynamic networks, we have excluded the realisations yielding no secondary infections^20^. This means that the actual average prevalence would be lower than the results shown in our figures, possibly worsen the agreement between the IBA and simulations. To extend the IBA to account for the absorption probability (epidemic spreading terminates at time *t*) may be a useful improvement. It is not difficult to calculate the probability that no secondary infection happens.

The present study is limited to SIR epidemics, which is not necessarily the optimal model for various infectious diseases, and to relatively small contact networks, missing, for example, seasonal variations in human interactions or other state compartments. Future studies should investigate the generalisability of our framework to other epidemiological scenarios, particularly taking into account infections with time scales larger than the ones studied here, such as measles and mumps, and models with more compartments. For example, adding a latent (or exposed, often denoted by *E*) compartment, unable to generate infections, would request a few changes to equations (3, 4, 5) and the addition of a new equation to represent the evolution of *E*(*t*), whereas equation (2) would be unaltered.

## Methods

We provide a description of the data sets used for the analysis in the main text and the protocol for the numerical simulations on dynamic networks. In the Supplementary Information, we derive the discretisation of the continuous-time IBA and analyse another data set to support our main conclusions.

### Network Data

We use data sets of dynamic contact networks representing face-to-face human interactions between delegates in a conference and visitors to a museum^29^. Each individual wore a wireless device such that a close face-to-face event was recorded every *T*_w_ = 20 sec. The conference, with *N* = 113 individuals, lasted for *T* ~ 59 hours, which gave *t*_max_ = 10,618. The museum visit, with *N* = 72 individuals, lasted for *T* ~ 7.29 hours, giving *t*_max_ = 1,311. The time of the first contact defines *t* = 0. The conference data set has *C* = 20,818 contacts between *E* = 2,196 unique pairs of individuals and the museum data set has *C* = 6,980 and *E* = 691. A third data set and the corresponding results are described in the Supplementary Information.

### Simulations on Dynamic Networks

We simulate the epidemic process directly in the original network data (S-DNO) and in its randomised version (S-DNR). In S-DNR, the daily cycles and network structure are conserved but the timing of each contact is uniformly randomised, i.e., the time-stamps of two links within a day (a day starts in the morning and finishes in the evening, starting/ending times estimated by the start/end of several subsequent contacts) are uniformly selected and swapped (we repeat the procedure twice the number of contacts within a day). Note that the museum dataset corresponds to a single day only.

In both S-DNO and S-DNR, at each time step, an infected individual may recover with probability *μ* and then infects, if still infective, each of its susceptible neighbours with probability *β*. We then, update the state of all nodes, measure them, and move to the next time step. For each infection seed, we take averages over 200 realisations.

To calculate the effective reproduction number using direct numerical simulations^35^,^48^, we start the infection at a given node (i.e. the index individual) and set the rest of the population to the susceptible state. We then let the infection evolves, as described above, and count the number of secondary infections caused by this index individual between times *t*_1_ + 1 and *t*_2_. For each index individual, we take the average over 200 realisations as the numerical estimation of *R*_eff_(*i*, *t*_1_, *t*_2_).

## Additional Information

**How to cite this article**: Rocha, L. E. C. and Masuda, N. Individual-based approach to epidemic processes on arbitrary dynamic contact networks. *Sci. Rep.* **6**, 31456; doi: 10.1038/srep31456 (2016).

## Supplementary Material

Supplementary Information

## References

[b1] Fonkwo P. N. Pricing infectious disease. The economic and health implications of infectious diseases. EMBO Rep. 9, S13–S17 (2008). 18578017 10.1038/embor.2008.110 PMC3327542

[b2] Keeling M. J. & Rohani P. Modeling infectious diseases Infectious Diseases in Human and Animals. (Princeton University Press, 2007).

[b3] Wallinga J., Edmunds W. J. & Kretzschmar M. Perspective: Human contact patterns and the spread of airborne infectious diseases. Trends Microbiol. 7, 372–377 (1999). 10470046 10.1016/s0966-842x(99)01546-2

[b4] Danon L. . Networks and the epidemiology of infectious disease. Interdiscip. Perspect. Infect. Dis. 1–28 (2011). 10.1155/2011/284909 PMC3062985 21437001

[b5] Holme P. & Saramäki J. Temporal networks. Phys. Rep. 519, 97–125 (2012).

[b6] Keeling M. J. & Eames K. T. D. Networks and epidemic models. J. R. Soc. Interface 2, 295–307 (2005). 16849187 10.1098/rsif.2005.0051 PMC1578276

[b7] May R. M. Network structure and the biology of populations. Trends Ecol. Evol. 21, 394–399 (2006). 16815438 10.1016/j.tree.2006.03.013

[b8] Pastor-Satorras R., Castellano C., Van Mieghem P. & Vespignani A. Epidemic processes in complex networks. Rev. Mod. Phys. 87, 925–979 (2015).

[b9] Volz E. & Meyers L. A. SIR epidemics in dynamic contact networks. Proc. R. Soc. B 274, 1628 (2007). 10.1098/rspb.2007.1159 PMC2291166 17878137

[b10] Morris M. & Kretzschmar M. Concurrent partnerships and the spread of HIV. AIDS 5, 641–648 (1997). 9108946 10.1097/00002030-199705000-00012

[b11] Bansal S., Read J., Pourbohloul B. & Meyers L. A. The dynamic nature of contact networks in infectious disease epidemiology. J. Biol. Dyn. 4, 478–489 (2010). 22877143 10.1080/17513758.2010.503376

[b12] Scholtes I. . Causality-driven slow-down and speed-up of diffusion in non-Markovian temporal networks. Nat. Comm. 5, 5024 (2014). 10.1038/ncomms6024 25248462

[b13] Delvenne J.-C., Lambiotte R. & Rocha L. E. C. Diffusion on networked systems is a question of time or structure. Nat. Comm. 6, 7366 (2015). 10.1038/ncomms8366 26054307

[b14] Wang Y., Chakrabarti D., Wang C. & Faloutsos C. Epidemic spreading in real networks: An eigenvalue viewpoint. Proc. 22nd Int. Symp. Rel. Dist. Sys. (SRDS’03) 25–34 (2003).

[b15] Draief M. Epidemic processes on complex networks. Phys. A 120–131 (2006).

[b16] Van Mieghem P., Omic J. & Kooij R. Virus spread in networks. IEEE Trans. Net. 17, 1–14 (2009).

[b17] Castellano C. & Pastor-Satorras R. Thresholds for epidemic spreading in networks. Phys. Rev. Lett. 105, 218701 (2010). 21231361 10.1103/PhysRevLett.105.218701

[b18] Gómez S., Arenas A., Borge-Holthoefer J., Meloni S. & Moreno Y. Discrete-time Markov chain approach to contact-based disease spreading in complex networks. EPL 89, 38009 (2010).

[b19] Draief M., Ganesh A. & Massoulie L. Thresholds for virus spread on networks. Ann. Appl. Prob. 18, 359–378 (2008).

[b20] Sharkey K. J. Deterministic epidemiological models at the individual level. J. Math. Biol. 57, 311–331 (2008). 18273619 10.1007/s00285-008-0161-7

[b21] Sharkey K. J. Deterministic epidemic models on contact networks: Correlations and unbiological terms. Theor. Popul. Biol. 79, 115–129 (2011). 21354193 10.1016/j.tpb.2011.01.004

[b22] Youssef M. & Scoglio C. An individual-based approach to SIR epidemics in contact networks. J. Theor. Biol. 283, 136–144 (2011). 21663750 10.1016/j.jtbi.2011.05.029

[b23] Guo D., Trajanovski S., van de Bovenkamp R., Wang H. & Van Mieghem P. Epidemic threshold and topological structure of susceptible-infectious-susceptible epidemics in adaptive networks. Phys. Rev. E 88, 042802 (2013). 10.1103/PhysRevE.88.042802 24229221

[b24] Valdano E., Ferreri L., Poletto C. & Colizza V. Analytical computation of the epidemic threshold on temporal networks. Phys. Rev. X 5, 021005 (2015).

[b25] Brauer F. Mathematical Epidemiology (Springer-Verlag, 2008).

[b26] Galvani A. P. & May R. M. Epidemiology: Dimensions of superspreading. Nature 438, 293–295 (2005). 16292292 10.1038/438293a PMC7095140

[b27] Antulov-Fantulin N., Lancic A., Smuc T., Stefancic H. & Sikic M. Identification of patient zero in static and temporal networks: Robustness and limitations. Phys. Rev. Lett. 114 248701 (2015). 26197016 10.1103/PhysRevLett.114.248701

[b28] How long is someone infectious after a viral infection? *National Health Service, England*, www.nhs.uk (2015). (Date of access:04/05/2016).

[b29] Isella L. . What’s in a crowd? Analysis of face-to-face behavioral networks. J. Theo. Biol. 271, 166–180 (2011). 10.1016/j.jtbi.2010.11.033 21130777

[b30] Lloyd-Smith J. O., Schreiber S. J., Kopp P. E. & Getz W. M. Superspreading and the effect of individual variation on disease emergence. Nature 438, 355–359 (2005). 16292310 10.1038/nature04153 PMC7094981

[b31] Kitsak M. . Identification of influential spreaders in complex networks. Nat. Phys. 6, 888–893 (2010).

[b32] Cross P. C., Lloyd-Smith J. O., Johnson P. L. F. & Getz W. M. Duelling timescales of host movement and disease recovery determine invasion of disease in structured populations. Ecol. Lett. 8, 587–595 (2005).

[b33] Heffernan J. M., Smith R. J. & Wahl L. M. Perspectives on the basic reproductive ratio. Clin. Infect. Dis. 2, 281–293 (2005). 10.1098/rsif.2005.0042 PMC1578275 16849186

[b34] Fine P., Eames K. & Heymann D. L. “Herd immunity”: A rough guide. Clin. Infect. Dis. 52, 911–916 (2011). 21427399 10.1093/cid/cir007

[b35] Rocha L. E. C. & Blondel V. D. Bursts of vertex activation and epidemics in evolving networks. PLOS Comput. Biol. 9, e1002974 (2013). 23555211 10.1371/journal.pcbi.1002974 PMC3605099

[b36] Karsai M. . Small but slow world: How network topology and burstiness slow down spreading. Phys. Rev. E 83, 025102(R) (2011). 10.1103/PhysRevE.83.025102 21405879

[b37] Timmreck T. C. An Introduction to Epidemiology. (Jones & Bartlett, 2002).

[b38] Shah D. & Zaman T. Detecting sources of computer viruses in networks: theory and experiment. In Proc. SIGMETRICS’10 203–214 (2010).

[b39] Pinto P. C., Thiran P. & Vetterli M. Locating the source of diffusion in large-scale networks. Phys. Rev. Lett. 109 068702 (2012). 23006310 10.1103/PhysRevLett.109.068702

[b40] Brockmann D. & Helbing D. The hidden geometry of complex, network-driven contagion phenomena. Science 342, 1337–1342 (2013). 24337289 10.1126/science.1245200

[b41] Salathé M. . A high-resolution human contact network for infectious disease transmission. Proc. Natl. Acad. Sci. USA 107, 22020–22025 (2010). 21149721 10.1073/pnas.1009094108 PMC3009790

[b42] Vanhems P. . Estimating potential infection transmission routes in hospital wards using wearable proximity sensors. PLOS ONE 8, e73970 (2013). 24040129 10.1371/journal.pone.0073970 PMC3770639

[b43] Bootsma M. C. J., Diekmann O. & Bonten M. J. M. Controlling methicillin-resistant Staphylococcus aureus: Quantifying the effects of interventions and rapid diagnostic testing. Proc. Natl. Acad. Sci. USA 103, 5620–5625 (2006). 16565219 10.1073/pnas.0510077103 PMC1459403

[b44] Hollingsworth T. D., Ferguson N. M. & Anderson R. M. Will travel restrictions control the international spread of pandemic influenza? Nat. Med. 12, 497–499 (2006). 16675989 10.1038/nm0506-497

[b45] Bajardi P. . Human mobility networks, travel restrictions, and the global spread of 2009 H1N1 pandemic. PLOS ONE 6, e16591 (2011). 21304943 10.1371/journal.pone.0016591 PMC3031602

[b46] Christakis N. A. & Fowler J. H. Social network sensors for early detection of contagious outbreaks. PLOS ONE 5, e12948 (2010). 20856792 10.1371/journal.pone.0012948 PMC2939797

[b47] Bajardi P., Barrat A., Savini L. & Colizza V. Optimizing surveillance for livestock disease spreading through animal movements. J. R. Soc. Interface 9, 2814–2825 (2012). 22728387 10.1098/rsif.2012.0289 PMC3479905

[b48] Holme P. & Masuda N. The basic reproduction number as a predictor for epidemic outbreaks in temporal networks. PLOS ONE 10, e0120567 (2015). 25793764 10.1371/journal.pone.0120567 PMC4368036

