# Supplementary material for: Individual-based approach to epidemic processes on arbitrary dynamic contact networks
Source: Sci Rep. 2016 Aug 26;6:31456. doi: 10.1038/srep31456 (PMC4999888; doi:10.1038/srep31456)
Supplement: Supplementary Information [file srep31456-s1.pdf]

Supplementary Information: Individual-based approach to epidemic processes on arbitrary dynamic contact network

Luis E C Rocha*

Department of Mathematics and naXys, Université de Namur, Namur, Belgium
Department of Public Health Sciences, Karolinska Institutet, Stockholm, Sweden

Naoki Masuda†

Department of Engineering Mathematics, University of Bristol, Bristol, UK

(Dated: June 10, 2016)

1. Discretisation of the continuous-time IBA

The IBA to the SIR dynamics on static networks in continuous time has been known[1–3]. By translating it to dynamic contact networks, we obtain the following approximate deterministic dynamics in continuous time:

$$\frac{\mathrm{d}S_i(t)}{\mathrm{d}t} = -S_i(t)\beta' \sum_{j\in\mathcal{N}_i(t)} I_j(t), \quad (1)$$

$$\frac{\mathrm{d}I_i(t)}{\mathrm{d}t} = S_i(t)\beta' \sum_{j\in\mathcal{N}_i(t)} I_j(t) - \mu' I_i(t), \quad (2)$$

$$\frac{\mathrm{d}R_i(t)}{\mathrm{d}t} = \mu' I_i(t), \quad (3)$$

where β' and μ' are the infection and recovery rates, respectively. We distinguish them from the infection probability β and the recovery probability μ in the main text because we assume discrete time in the IBA, whereas Equations (1), (2), and (3) assume continuous time.

We discretise equations (1), (2), and (3) with a time step Δt to obtain

$$S_i(t) = S_i(t-\Delta t) - S_i(t-\Delta t)(\beta'\Delta t) \sum_{j\in\mathcal{N}_i(t-\Delta t)} I_j(t-\Delta t), \quad (4)$$

$$I_i(t) = I_i(t-\Delta t) + S_i(t-\Delta t)(\beta'\Delta t) \sum_{j\in\mathcal{N}_i(t-\Delta t)} I_j(t-\Delta t) - (\mu'\Delta t) I_i(t-\Delta t), \quad (5)$$

$$R_i(t) = R_i(t-\Delta t) + (\mu'\Delta t) I_i(t-\Delta t). \quad (6)$$

The time discretisation is justified when the probabilities of state-transition events within Δt are sufficiently small. In the present case, this is equivalent to saying $\beta'\Delta t, \mu'\Delta t \ll 1$. By assuming that Δt is the duration of the single snapshot of temporal networks, we obtain $\beta = \beta'\Delta t$ and $\mu = \mu'\Delta t$. Because our discrete-time approach is justified only when $\beta, \mu \ll 1$, the time discretisation of the continuous-time SIR model is consistent with the assumption justifying our discrete-time approach.

To make an intuitive understanding and comparison with the IBA easier, we change from the continuous-time to discrete-time notation and replace $t - \Delta t$ by $t - 1$:

$$S_i(t) = S_i(t-1) - S_i(t-1)\beta \sum_{j\in\mathcal{N}_i(t-1)} I_j(t-1), \quad (7)$$

$$I_i(t) = I_i(t-1) + S_i(t-1)\beta \sum_{j\in\mathcal{N}_i(t-1)} I_j(t-1) - \mu I_i(t-1), \quad (8)$$

$$R_i(t) = R_i(t-1) + \mu I_i(t-1). \quad (9)$$

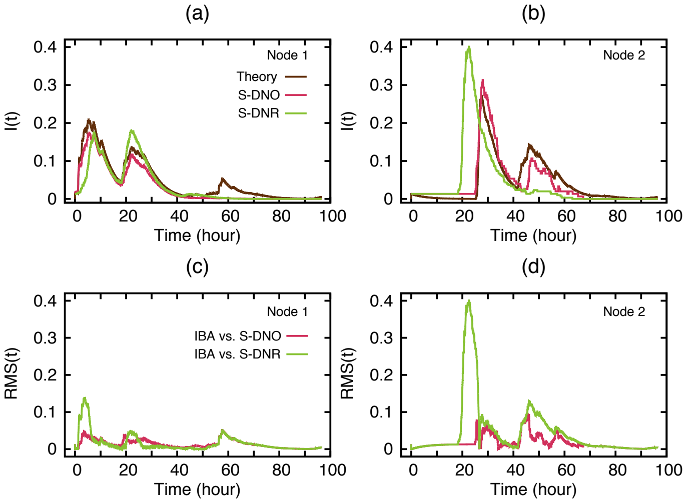

Supplementary Figure 1 Accuracy of different approximation methods. (a)–(b) The fraction of infected individuals (I) as a function of time (i.e. the prevalence $I(t)$) for $\beta = 0.1$ and $\mu = 0.001$ using the hospital data set. (c)–(d) Root-mean-square (RMS) deviation between the IBA and S-DNO, and the IBA and S-DNR.

If we expand equations (3) and (4) in the main text in terms of small parameters β and μ, and only retain the first-order terms, we obtain the variants of equations (7) and (8), where $\mathcal{N}_i(t-1)$ in equations (7) and (8) is replaced by $\mathcal{N}_i(t)$. This minor difference arose because, in equations (4) and (5), we used the snapshot network at $t - \Delta t$ to evolve the dynamics, whereas the snapshot network at t is used to

*Electronic address: luis.rocha@ki.se
†Electronic address: naoki.masuda@bristol.ac.uk

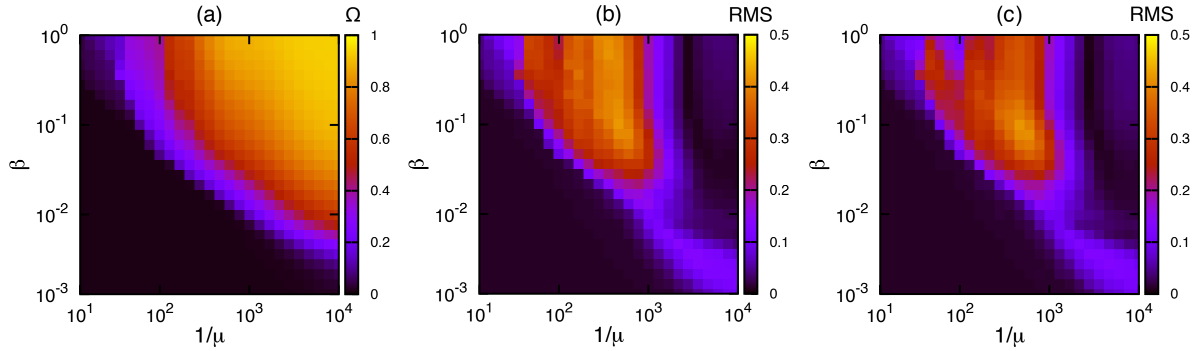

Supplementary Figure 2 Final outbreak size. (a) Final outbreak size $\Omega \equiv R(t_{\max}) + I(t_{\max})$ for the IBA. We take averages over all individuals as seeds. The colours represent the values of Ω. Root-mean-square deviation (RMS) between (b) the IBA and S-DNO, and (c) the IBA and S-DNR. For the S-DNO and S-DNR cases, we remove null and small ($< 0.1N$) outbreaks to calculate Ω. The colours represent the root-mean-square deviation between the respective two approaches.

evolve the dynamics in equations (3) and (4) in the main text.

2. The hospital dynamic contact network

We carried out the analyses presented in the main text to a third real-life dynamic contact network to assess the generalisability of the results. This data set corresponds to face-to-face human interaction between patients and health-care workers in a hospital ward[4]. The network contains $N = 75$ nodes, $E = 1,139$ unique pairs of contacts and $C = 32,424$ temporal contacts distributed in $T = 96.57$ hours, giving $t_{\max} = 17,382$. As we show in the following, the results obtained for the conference and museum data sets, used in the main text, qualitatively hold true for this data set as well.

2.1. Number of infected individuals

Figure S1(a,b) shows the evolution of the fraction of infected individuals, $I(t)$ (i.e. the prevalence) given two different initial conditions (i.e. different seeds). We compare the estimation of the prevalence based on the IBA and the simulations on the original empirical network and its randomised version. In line with the results for the conference and museum data sets shown in the main text, we observe a reasonably good agreement between the IBA and S-DNO, including the reproduction of both peaks. On the other hand, the mismatch between the IBA and the S-SNR is evident, particularly for node 2, in which the peak prevalence is shifted to earlier times. This is a consequence of the uniformly distributed contacts after randomisation. By calculating the root-mean-square between the models Figure S1(c,d), we confirm the qualitative observation that IBA better captures the original contact sequence.

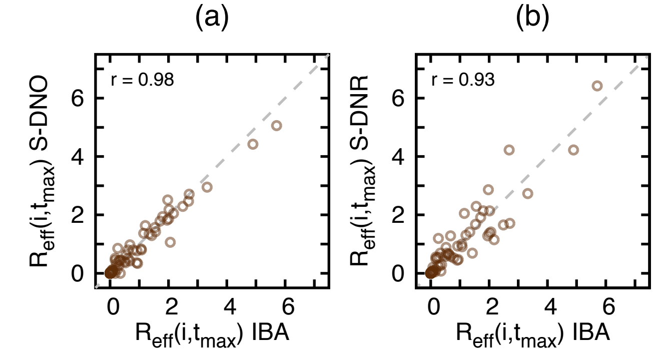

Supplementary Figure 3 Individual effective reproduction number. (a) Comparison between the IBA and S-DNO. (b) Comparison between the IBA and S-DNR. Each of the $N = 75$ circles represents an index individual i. Values are estimated at time $t_{\max}$ and the epidemiological parameters are set to $\beta = 0.1$ and $\mu = 0.001$. The Pearson correlation coefficient is denoted by r.

2.2. Final outbreak size

Figure 2 shows the final outbreak size calculated using the IBA approximation. We observe that results are similar for both simulation methods S-DNO (Fig. S2(b)) and S-DNR (Fig. S2(c)). This result indicates that for this particular dataset, randomization of the time-stamps yields little difference in the final outbreak. As we can see in Fig. S1(a,b), though with different shapes, both simulation methods may infect approximately the same number of nodes because a higher infection in the first wave balances a relatively smaller infection in the second wave.

2.3. Individual reproduction number

The IBA is also accurate in approximating the S-DNO in terms of the individual reproduction number (Fig. S3(a,b)). The agreement is stronger between the IBA and S-DNO than between the IBA and S-DNR, as observed in the other data sets. The correlation coefficients are larger for the hospi-

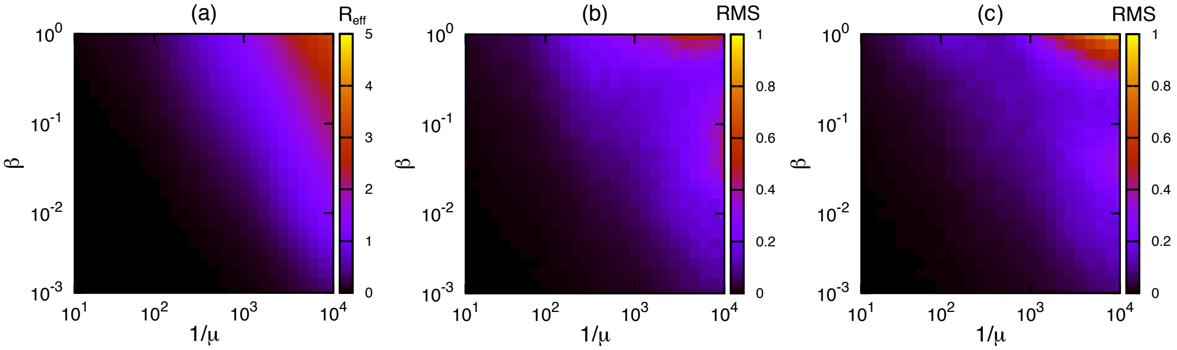

Supplementary Figure 4 **Effective reproduction number.** (a) Estimation of the effective reproduction number $R_{\rm eff}(t_{\rm max})$ using the IBA. The colours represent the value of $R_{\rm eff}(t_{\rm max})$. Root-mean-square deviation between the (b) IBA and S-DNO and (c) the IBA and S-DNR for various epidemiological parameters β and μ. The colours in (b)–(c) represent the root-mean-square deviation. See Materials and Methods in the main text for details on the numerical calculations.

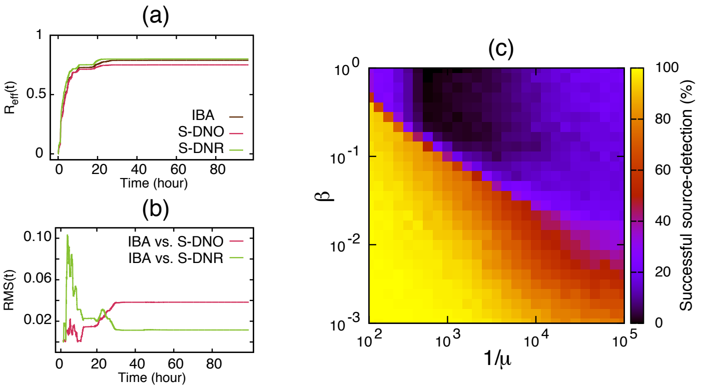

Supplementary Figure 5 **Time dependence of the effective reproduction number and source detection.** (a) Time dependence of the effective reproduction number $R_{\rm eff}(t)$ calculated using different models with $\beta = 0.1$ and $\mu = 0.001$ for the hospital data set. (b) Root-mean-square (RMS) difference between the IBA and S-DNO, and IBA and S-DNR for the hospital data set. See Materials and Methods in the main text for details on the calculations. (c) Percentage of successfully detected infection sources (estimated on the basis of 1000 randomly located sources) for various β and μ values for the hospital data set.

tal data set than for the conference and museum data sets presented in the main text.

2.4. Effective reproduction number

We calculate the effective reproduction number for the IBA (Fig. S4(a)), S-DNO and S-DNR models. By calculation of the root-mean-square, we observe that the results agree between the IBA and S-DNO (Fig. S4(b)) and between IBA and S-DNR (Fig. S4(c)) in the full range of parameters studied. This good agreement is possibly a result of the range of parameters studied. More significant differences show up for smaller values of μ, as seen on the top right part of Fig. S4(c).

2.5. Source detection

We perform the source-detection experiments using the hospital data set. Similarly to results for the conference and museum data sets, the IBA efficiently detects the source of infection given the states of individuals at time $t_{\rm max}$ and the past contact patterns (Fig. S5).

References

[1] Sharkey, K. J. Deterministic epidemiological models at the individual level. *J. Math. Biol.* **57** 311–331 (2008).

[2] Sharkey, K. J. Deterministic epidemic models on contact networks: Correlations and unbiological terms. *Theor. Popul. Biol.* **79** 115–129 (2011).

[3] Youssef, M. & Scoglio, C. An individual-based approach to SIR epidemics in contact networks. *J. Theor. Biol.* **283** 136–144 (2011).

[4] Vanhems, P., et al. Estimating potential infection transmission routes in hospital wards using wearable proximity sensors. *PLOS ONE* **8** e73970 (2013).
